# Phosphorothioate oligonucleotides, suramin and heparin inhibit DNA-dependent protein kinase activity

**DOI:** 10.1038/sj.bjc.6600191

**Published:** 2002-04-08

**Authors:** Y Hosoi, Y Matsumoto, M Tomita, A Enomoto, A Morita, K Sakai, N Umeda, H-J Zhao, K Nakagawa, T Ono, N Suzuki

**Affiliations:** Department of Radiation Oncology, Faculty of Medicine, University of Tokyo, Tokyo, Japan; Department of Radiology, Faculty of Medicine, University of Tokyo, Tokyo, Japan; Department of Radiation Research, Tohoku University School of Medicine, Sendai, Japan; Low Dose Radiation Research Center, Central Research Institute of Electric Power Industry, Tokyo, Japan

**Keywords:** DNA-PK, phosphorothioate oligonucleotides, suramin, radiation sensitivity, DNA double-stranded breaks

## Abstract

Phosphorothioate oligonucleotides and suramin bind to heparin binding proteins including DNA polymerases, and inhibit their functions. In the present study, we report inhibition of DNA-dependent protein kinase activity by phosphorothioate oligonucleotides, suramin and heparin. Inhibitory effect of phosphorothioate oligonucleotides on DNA-dependent protein kinase activity was increased with length and reached a plateau at 36-mer. The base composition of phosphorothioate oligonucleotides did not affect the inhibitory effect. The inhibitory effect by phosphorothioate oligodeoxycytidine 36-mer can be about 200-fold greater than that by the phosphodiester oligodeoxycytidine 36-mer. The inhibitory effect was also observed with purified DNA-dependent protein kinase, which suggests direct interaction between DNA-dependent protein kinase and phosphorothioate oligonucleotides. DNA-dependent protein kinase will have different binding positions for double-stranded DNA and phosphorothioate oligodeoxycytidine 36-mer because they were not competitive in DNA-dependent protein kinase activation. Suramin and heparin inhibited DNA-dependent protein kinase activity with IC_50_ of 1.7 μM and 0.27 μg ml^−1^ respectively. DNA-dependent protein kinase activities and DNA double-stranded breaks repair in cultured cells were significantly suppressed by the treatment with suramin *in vivo*. Our present observations suggest that suramin may possibly result in sensitisation of cells to ionising radiation by inactivation of DNA-dependent protein kinase and the impairment of double-stranded breaks repair.

*British Journal of Cancer* (2002) **86**, 1143–1149. DOI: 10.1038/sj/bjc/6600191 www.bjcancer.com

© 2002 Cancer Research UK

## 

Phosphorothioate oligonucleotide (S-oligo) is an analogue of phosphodiester oligonucleotide having modified internucleotide linkages that make it more stable to nucleases than phosphodiester oligonucleotide (Wagner, 1995). Antisense S-oligo is designed to have the complementary base sequence for binding the target mRNA specifically. However, the ability of S-oligo to bind non-specifically to a variety of proteins has been well documented (Yakubov *et al*, 1993; Stein 1995). Such proteins include heparin-binding proteins such as basic fibroblast growth factor (bFGF), acid fibroblast growth factor (aFGF), Kaposi's growth factor (FGF-4) (Guvakova *et al*, 1995), and DNA polymerases (Gao *et al*, 1992). S-oligo also inhibits the functions of other proteins such as CD4 (Yakubov *et al*, 1993), gp120 (Stein *et al*, 1991), Mac-1 (Benimetskaya *et al*, 1997), RNase H (Gao *et al*, 1992), human immunodeficiency virus type 1 (HIV-1) reverse transcriptase (Marshall *et al*, 1992), herpes simplex virus (HSV) type 2-induced DNA polymerase (Gao *et al*, 1989), and HIV-1 integrase (Tramontano *et al*, 1998).

Suramin has been used as an anti-cancer agent and an anti-HIV agent (Stein, 1993). The structure of suramin is shown in Figure 1

**Figure 1:**
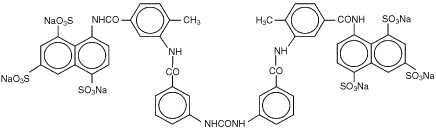
Structure of suramin. The hexasodium salt is shown. The molecular weight of suramin is 1429.

. Potential mechanisms of the anti-tumour effect are inhibition of heparin binding growth factors. Suramin binds to PDGF, basic fibroblast growth factor (bFGF) and other growth factors, and it prevents the binding of growth factors to their corresponding receptors (Hosang, 1985; Coffey *et al*, 1987; Stein, 1993). Potential mechanisms of the anti-HIV effect are inactivation of reverse transcriptase and DNA polymerases (Mitsuya *et al*, 1984; Spigelman *et al*, 1987; Stein, 1993). Suramin is also an inhibitor of Protein kinase C and DNA topoisomerase II (Mahoney *et al*, 1990; Bojanowski *et al*, 1992).

Thus both S-oligo and suramin act as heparin mimetics and they inhibit HIV-1 integration into host DNA (Stein *et al*, 1993; Benimetskaya *et al*, 1995). In the present study, we investigated the effects of S-oligo, suramin and heparin on activity of DNA-dependent protein kinase (DNA-PK) because DNA-PK is thought to be a heparin binding protein (Lees-Miller and Anderson, 1991) and because it is involved in the retroviral integration into host DNA (Daniel *et al*, 1999). We demonstrate that S-oligo, suramin and heparin inhibit DNA-PK activity. DNA-dependent protein kinase activities and DNA-double stranded breaks (DSBs) repair of cultured cells were significantly suppressed by the treatment with suramin. Our present observations suggest that suramin may possibly result in sensitisation of cells to ionising radiation by inactivation of DNA-PK and the impairment of DSBs repair.

## MATERIALS AND METHODS

### Cell lines

LM217 is an SV-40 transformed human fibroblast cell line derived from HS27 (Murnane, 1986), and it was obtained from Dr JP Murnane. A human glioblastoma cell line T98G, and a human acute lymphoblastic leukaemia cell line MOLT-4 were obtained from the ATCC (Rockville, MD, USA).

### Chemicals

Suramin was purchased from Calbiochem-Novabiochem Co. (La Jolla, CA, USA). Oligonucleotides were synthesised by standard phosphoramidite chemistry (Hokkaido System Science Co., Sapporo, Japan). The oligonucleotides were base-deblocked in 30% ammonium hydroxide at 55°C for 8 h and purified by reversed phase high pressure liquid chromatography (HPLC) (0.1 M triethylamine). The oligonucleotides were detritylated in 80% acetic acid, lyophilised and resuspended in 50% diethyl ether. The aqueous phase was recovered and the oligonucleotides were precipitated. Oligonucleotide concentrations were determined by spectroscopy. The sizes of oligonucleotides were confirmed by polyacrylamide gel electrophoresis. Phosphorothioate oligodeoxycytidine with different chain length (S-dCn), phosphorothioate oligodeoxyguanosine 36-mer (S-dG_36_), phosphorothioate oligodeoxythymidine 36-mer (S-dT_36_), phosphorothioate oligodeoxyadenosine 36-mer (S-dA_36_), and phosphodiester oligodeoxycytidine 36-mer (dC_36_) were synthesised.

### Digestion of S-dC_36_

For digestion of S-dC_36_, it was incubated in 2N HCl at 95°C for 1 h. After incubation, pH of the solution was adjusted to pH 7.2 using NaOH. The effect of the HCl-treatment was confirmed by polyacrylamide gel electrophoresis and no obvious band was observed.

### DNA-PK purification

DNA-dependent protein kinase was purified as described previously (Matsumoto *et al*, 1997). MOLT-4 cell nuclei were prepared from 2–5×10^9^ cells as described by Dignam *et al* (1983). The nuclei were resuspended in buffer A (20 mM HEPES-NaOH, pH 7.9; 400 mM KCl; 1 mM EDTA; 1 mM EGTA; 0.02% Tween 20; 10% glycerol; 1 mM dithiothreitol (DTT); 1 mM phenylmethylsulfonyl fluoride (PMSF); 1 μg ml^−1^ of leupeptin, pepstatin and antipain, respectively) and agitated with a stirring bar for 30 min followed by centrifugation at 100 000 **g** for 60 min. The supernatant nuclear extract was passed through the first DEAE Bio-Gel A (Bio-Rad Laboratories, Hercules, CA, USA) column and dialysed against buffer B (20 mM Tris-HCl, pH 7.5; 1 mM EDTA; 10% glycerol; 50 mM NaCl; 1 mM DTT; 1 mM PMSF; 1 μg ml^−1^ leupeptin; 1 μg ml^−1^ pepstatin; 1 μg ml^−1^ antipain). Dialysate was applied to the second DEAE Bio-Gel A and eluted with buffer B with increasing NaCl concentration linearly from 0.05 to 0.3 M. DNA-dependent protein kinase was eluted with 0.14–0.17 M NaCl. The DNA-PK fractions were passed through NAP-25 column (Amersham Pharmacia Biotech, Uppsala, Sweden) equilibrated with buffer C (20 mM HEPES-NaOH, pH 7.2; 1 mM MgCl_2_; 15% glycerol; 200 mM NaCl; 1 mM DTT; 1 mM PMSF; 1 μg ml^−1^ leupeptin; 1 μg ml^−1^ pepstatin, 1 μg ml^−1^ antipain) and finally loaded to a native DNA-cellulose column (Amersham Pharmacia Biotech). Absorbed protein was eluted into 12 fractions (1 ml each) by stepwise increase of NaCl concentration in buffer C. We used 0.6 M NaCl eluate as the purified DNA-PK holoenzyme. Final protein concentration of purified DNA-PK solution was 0.4 mg ml^−1^.

### DNA-PK activity measurement

DNA-dependent protein kinase activity was assayed as previously described using a synthetic peptide (EPPLSQEAFADLWKK) (Hosoi *et al*, 1998b). Whole cell extracts were prepared as described previously (Hosoi *et al*, 1998a). The cell extracts were incubated in 20 μl of kinase buffer (20 mM HEPES-NaOH, pH 7.2; 100 mM KCl; 5 mM MgCl_2_; 1 mM DTT; 0.5 mM NaF; 0.5 mM β-glycerophosphate; 0.2 mM ATP; 10 μCi ml^−1^ [γ-^32^P]ATP in the presence of 0.5 μg ml^−1^ Poly(dG-dC)·Poly(dG-dC) (Amersham Pharmacia Biotech) and 0.5 mg ml^−1^ substrate peptide) at 37°C for 15 min. The final protein concentration in the reaction buffer was 0.15 mg ml^−1^. In DNA-PK activity measurement using the purified DNA-PK solution, 0.25 μl of the solution was used in each reaction. The reactions were stopped by the addition of 20 μl of 30% acetic acid and spotted onto P81 paper disks (Whatman International Ltd., Maidstone, UK). The disks were washed four times in 15% acetic acid. Radioactivity in the paper disks was measured in a liquid scintillation counter. The DNA-PK activity is defined as the amount of P transferred from ATP to the synthetic peptide in the reaction. The specific activities of the cell extracts from LM217 cells, those from T98G cells and the purified DNA-PK solution were 19.0±2.7, 72.31±9.48 and 2316.0±128.9 pmol μg^−1^ protein respectively.

### Preparation of plug and irradiation

Cells in dishes were irradiated on ice using an X-ray machine SHIMADZU HF350C (Shimadzu Corporation, Kyoto, Japan) at 200 kV, 20 mA with a 0.5 mm Cu and 1.0 mm Al filter at a dose rate of 1.5 Gy min^−1^, and then incubated in a CO_2_ incubator at 37°C for repair. At each paoin, cells were trypsinised and washed two times with cold PBS, and the resulting cell pellet was embedded in 0.75% agarose (Low Melt Preparative Grade Agarose, Bio-Rad Laboratories). These agarose sample plugs were immersed in ice-cold lysis solution (0.5 M EDTA; 0.01 M Tris; 2% Sarcosyl; 0.2 mg ml^−1^ proteinase K) for 1 h and then incubated at 50°C for 48 h. After lysis, sample plugs were washed for 1 h at room temperature in a buffer containing 10 mM Tris (pH 8.0) and 0.1 M EDTA and then treated for 1 h at 37°C in the same buffer with 0.1 mg ml^−1^ RNase A (Wang *et al*, 2001). For the initial time (0 h), cells were embedded in agarose and irradiated in ice-cold PBS followed by an immediate lysis as described (Okayasu *et al*, 1998).

### Pulsed-field gel electrophoresis (PFGE)

Sample plugs were electrophoresed in 0.5×TBE buffer (45 mM Tris; 45 mM boric acid; 1.5 mM EDTA, pH 8.2) in a clamped homogenous electric field (CHEF) gel box (CHEF-DR® III System, Bio-Rad Laboratories) in 0.8% agarose gel (SeaKem GTG® agarose, Bio Whittaker Molecular Applications, ME, USA) at 14°C. The voltage applied was 6.0 V/cm with a 60-s pulse time for the first 9 h followed by 120-s pulse time for the last 15 h (total run time 24 h) (Okayasu *et al*, 1998). After electrophoresis, the gels were stained with ethidium bromide (1.0 mg ml^−1^), detained in deionised distilled water (Longo *et al*, 1997). The images were acquired under UV light using Printgraph AE-6910CX and ImageSaver AE-6905C (ATTO Corporation, Tokyo, Japan) and analysed by NIH image. Fraction of DNA in the lane (FDL) was calculated as [(average density in the lane)×(area of the lane)]/[(average density in the lane)×(area of the lane)+(average density in the well)×(area of the well)]. The background FDL, which corresponds to FDL without irradiation, was subtracted from each point.

## RESULTS

### Inhibition of DNA-PK activity was dependent on the chain length of S-dCn

**Figure 2:**
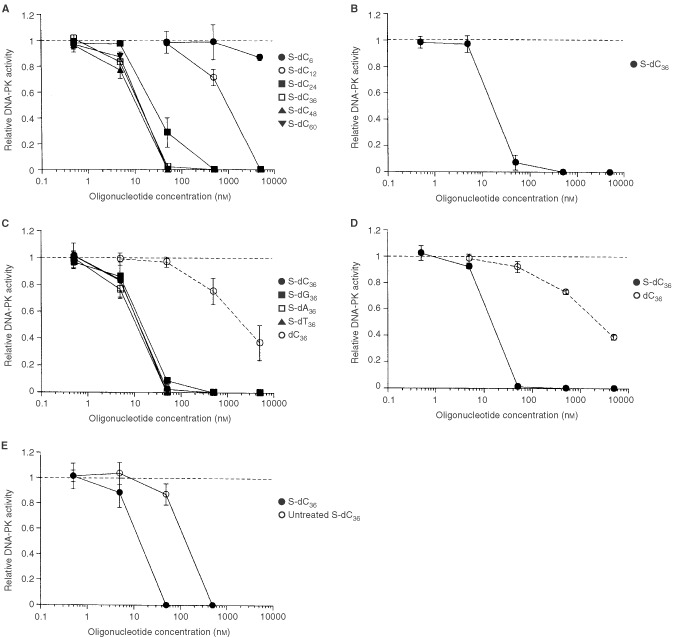
Effect of oligonucleotides on DNA-PK activity. (**A**) Effect of S-dC_6_, S-dC_12_, S-dC_24_, S-dC_36_, S-dC_48_, and S-dC_60_ on DAN-PK activity. Whole cell extract prepared from LM217 was used. (**B**) Effect of S-dC_36_ on DNA-PK activity. Whole cell extract prepared from T98G was used. (**C**) Effect of S-dC_36_, S-dG_36_, S-dA_36_, S-dT_36_, and dC_36_ on DNA-PK activity. Whole cell extract prepared from LM217 was used. (**D**) Effect of S-dC_36_ and dC_36_ on DNA-PK activity. Purified DNA-PK was used. (**E**) Effect of HCl-treated S-dC_36_ and untreated S-dC_36_ on DNA-PK activity. Whole cell extract prepared from LM217 was used. Salt concentration of untreated S-dC_36_ was adjusted to that of HCl-treated S-dC_36_. DNA-PK activities are expressed as values relative to that of control, which is set to a value of 1. The data represents the means±s.d. (*n*=3).

**Figure 3:**
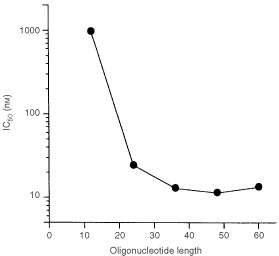
Dose-response curve after treatment with S-dCn. IC_50_ was calculated from the data used for Figure 2A.

DNA-dependent protein kinase activity was measured in the presence of S-dCn with different chain lengths. DNA-dependent protein kinase activity was inhibited by S-dCn depending on the concentration of S-dCn and the number of phosphorothioate linkages (Figure 2A

). Inhibitory concentration 50 (IC_50_) of S-dCn is shown in Figure 3

. The IC_50_ value decreased from 975 nM to 13 nM with the increased chain length from 12 to 36. The IC_50_ value reached a plateau with the 36-mer. To confirm whether this inhibitory effect was specific for LM217, the effect of S-dC_36_ on DNA-PK activity was investigated using whole cell extract derived from T98G. The IC_50_ in a T98G cell extract was almost the same as that in a LM217 cell extract (Figure 2B).

### Inhibition of DNA-PK activity was independent of the base composition

Next, effect of base composition on inhibition of DNA-PK activity was investigated. Effect of S-dC_36_, S-dG_36_, S-dA_36_, or S-dT_36_ on DNA-PK activity is shown in Figure 2C. Inhibition of DNA-PK activity was not affected by the base composition of phosphorothioate 36-mer oligonucleotides.

### Direct interaction between oligonucleotides and DNA-PK, and comparison between the effect of phosphodiester oligonucleotides on DNA-PK and that of phosphorothioate oligonucleotides

In order to confirm whether inhibition of DNA-PK activity by S-oligos is mediated by direct interaction with DNA-PK, purified DNA-PK was used. S-dC_36_ inhibited purified DNA-PK activity with the same IC_50_ as cell extracts, which is suggesting direct interaction between S-dC_36_ and DNA-PK (Figure 2D). Next, the effect of dC_36_ on DNA-PK was compared with the effect of S-dC_36_. DNA-dependent protein kinase activity was inhibited by dC_36_ (Figure 2C). However, dC_36_ showed much less inhibition than S-dC_36_. The IC_50_ of dC_36_ was about 200 times higher than that of S-dC_36_. Similar inhibition was observed with purified DNA-PK (Figure 2D).

### Inhibition of DNA-PK activity by S-dC_36_ was not due to chemical contaminants

The IC_50_ of S-dCn was not linearly proportional to the chain length (Figure 3), which suggests that the inhibition was not due to contaminants in the purified phosphorothioate oligonucleotides. To confirm this, S-dC_36_ was digested with HCl treatment at 95°C for 1 h and the effect of digested S-dC_36_ on DNA-PK was investigated. HCl-treatment decreased the IC_50_ value, which is indicating that the inhibition was not due to chemical contaminants (Figure 2E).

### S-dC_36_ did not compete with dsDNA for inhibition of DNA-PK activity

**Figure 4:**
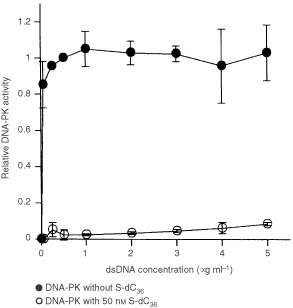
Effect of dsDNA concentration on inhibition of DNA-PK activity by S-dC_36_. Concentrations of dsDNA ranged from 0.05 μg ml^−1^ to 5 μg ml^−1^. Whole cell extract prepared from LM217 was used. Details are the same as shown in Figure 2. Figure 5. Effect of suramin (**A**) and heparin (**B**) on DNA-PK activity. Whole cell extract prepared from LM217 was used. Details are the same as shown in Figure 2.

Single-stranded DNA has been reported to inhibit DNA-PK activity by binding to DNA-PK at a different site from the dsDNA-binding site (Leuther *et al*, 1999). To gain insight into whether the S-dC_36_-binding site differs from the dsDNA-binding site, we investigated whether dsDNA competes with S-dC_36_ in DNA-PK activation. DNA-dependent protein kinase activation by dsDNA reached its maximal level at a concentration of 0.5 μg ml^−1^, remaining unchanged as the concentration increased up to 5 μg ml^−1^ (Figure 4

). Inhibition of DNA-PK activity by 50 nM S-dC_36_ was unaffected by change in dsDNA concentration (Figure 4). This non-competitive inhibition suggests that the S-dC_36_-binding site is distinct from the dsDNA-binding site.

### Inhibition of DNA-PK activity by suramin and heparin

**Figure 5:**
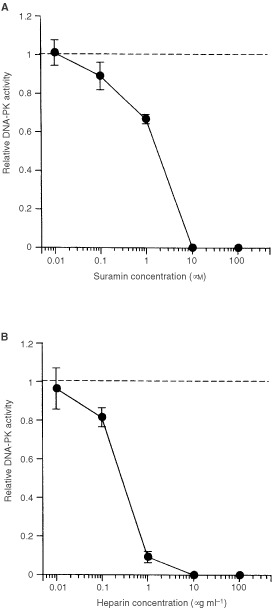
Effect of suramin (**A**) and heparin (**B**) on DNA-PK activity. Whole cell extract prepared from LM217 was used. Details are the same as shown in Figure 2.

Effects of suramin and heparin on DNA-PK activity in cell extracts are shown in Figure 5A,B

. Suramin and heparin suppressed DNA-PK activity with IC_50_ of 1.7 μM and 0.27 μg ml^−1^ respectively. Next, we investigated effects of suramin on DNA-PK activity in cultured cells. LM217 cells at growth phase were treated with 1 mM suramin for 20 h. After treatment, cells were washed with PBS three times, total cell extracts were prepared, and DNA-PK activities were measured. DNA-PK activities were significantly suppressed by the treatment with suramin to 80.7±8.7% of the control value (*P*=0.045). The treatment with 1 mM suramin for 24 h did not affect the plating efficiency of LM217 cells (data not shown).

### Inhibition of DNA repair by suramin

**Figure 6:**
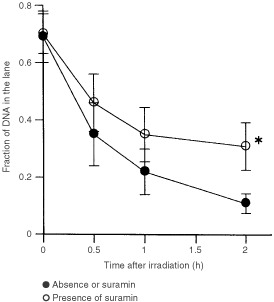
Kinetics of DNA double strand breaks after 50 Gy irradiation in exponentially growing LM217 cells in the presence or absence of 1 mM suramin. Cells were treated with suramin 20 h before irradiation and the drug was kept in the medium during the post-irradiation repair period. The data represent the means±s.d. (*n*=3). *P<0.05.

Because treatment with 1 mM suramin *in vivo* suppressed DNA-PK activity to 80.7% of the control value, we investigated the effect of suramin on DNA-repair after irradiation. After 20 h treatment with 1 mM suramin, LM217 cells were irradiated with 50 Gy, and DSBs repair was analysed by PFGE. The FDL is reported to be proportional to the ratio of fragmented double-stranded DNA (Okayasu *et al*, 1998). Although the initial DSBs (0 h) with 50 Gy irradiation was statistically identical for suramin-treated and untreated cells, the significant inhibition of DSBs repair was observed in samples treated with suramin (Figure 6

).

## DISCUSSION

S-oligos bind sequence-independently to a variety of proteins and inhibit their functions (Stein, 1995). S-oligos act as heparin mimetics and they bind to heparin binding proteins such as bFGF (Benimetskaya *et al*, 1995). Furthermore, S-oligos inhibit HIV-1 DNA integration (Matsukura *et al*, 1987; Agrawal *et al*, 1988). In these respects, S-oligos resemble such polyanions as suramin and pentosan polysulphate (Stein, 1995). Suramin and pentosan polysulphate also bind to heparin binding proteins and inhibit HIV-1 DNA integration (Mitsuya *et al*, 1984; Zugmaier *et al*, 1992; Stein, 1993). In the present study, we demonstrated that both S-oligos and suramin show the same effects on DNA-PK activity.

Binding of S-oligos to heparin binding proteins is sequence-independent but is dependent on the chain length of S-oligos. It has been reported that S-oligos with a chain length longer than 15 could bind and inhibit the proteins, and that the inhibitory effect reached a plateau level with a chain length of 28 (Gao *et al*, 1992; Marshall *et al*, 1992). In the present study, we reported that the inhibitory effect of S-oligos on DNA-PK activity is sequence-independent and is dependent on the chain length. The inhibitory effect reached a plateau level with a chain length of 36, which is almost the same length as previously reported. Marshall *et al* (1992) reported that the inhibitory effect of oligonucleotides on HIV-1 reverse transcriptase could be at least 30-fold greater with phosphorodithioate oligonucleotides, which have two sulphur at each site of internucleotide linkages, than with phosphorothioate oligonucleotides. Benimetskaya *et al* (1995) reported that binding of phosphorothioate oligonucleotides to proteins is independent on P-chirality at the internucleotide linkage sites. These results may suggest that substances having the similar structure to polyanions with sulphur bind heparin-binding proteins and inhibit their functions (Zugmaier *et al*, 1992; Guvakova *et al*, 1995).

Phosphorothioate oligonucleotides contain a sulphur atom at each phosphorus atom. Each *n*-mer phosphorothioate oligonucleotide has (*n*-1)-centres of asymmetry at phosphorous because each linkage can occur as either the Rp- of Sp-diastereomer (Benimetskaya *et al*, 1995). Phosphorothioate oligonucleotides used in this experiment contained a random mixture of diastereomers that would have a variety of three-dimensional structure. Benimetskaya *et al* (1995) reported that binding of phosphorothioate oligonucleotides to basic fibroblast growth factor, recombinant soluble CD4, laminin and fibronectin is P-chirality independent. It is unknown what effect the three-dimensional structure may have on the interactions of phosphorothioate oligonucleotides with DNA-PK.

Both S-oligos and suramin inhibit the binding of HIV-1 gp120 to CD40 and the enzyme activity of DNA polymerases, RNase H and HIV-1 integrase (Gao *et al*, 1989, 1992; Stein *et al*, 1991, 1993; Tramontano *et al*, 1998). All of these function and enzymes are related to HIV-1 infection and integration into host DNA. Recently, DNA-PK was reported to be involved in the retroviral integration into host DNA (Daniel *et al*, 1999). S-oligos and suramin will be potent anti-HIV-1 agents. In addition, it is interesting that most of the steps of HIV-1 integration into the host DNA are inhibited by S-oligos and suramin.

The mechanisms of DNA-PK inactivation by S-oligos and suramin are not known. Hammarsten and Chu (1998) reported that single-stranded DNA (ssDNA) did not inhibit the binding of dsDNA to Ku but it inhibits the binding of dsDNA to DNA-PK. The structure of DNA-PK was revealed by electron crystallography (Chiu *et al*, 1998; Leuther *et al*, 1999). Chiu *et al* (1998) reported that DNA-PKcs protein has an open, cage-like structure, which may allow the insertion of two DNA ends from the two opposing faces of the protein. Leuther *et al* (1999) reported that structure of DNA-PKcs protein contains an open channel, similar to those seen in other double-stranded DNA-binding proteins, and a cavity which is large enough to accommodate ssDNA. They suggest that ssDNA binds to the enclosed cavity and inhibit DNA-PK activity. These reports suggest that ssDNA binds to DNA-PK at a position different from where dsDNA binds to DNA-PK. In the present study, we reported that inhibition of DNA-PK activity by S-oligos is not competitive with dsDNA, suggesting that the binding position of S-oligos is different from that of dsDNA. Further experiments are required to elucidate this point.

Cells lacking DNA-PK activity due to defects in DNA-PK components show hypersensitivity to ionising radiation because of an important role of DNA-PK in the repair of DNA double-strand breaks (Jeggo *et al*, 1995). A phosphatidylinositol 3-kinase (PI3-kinase) inhibitor, wortmannin, inhibits DNA-PK activity, DSBs repair and sensitises cells to ionising radiation (Rosenzweig *et al*, 1997; Hosoi *et al*, 1998b). Suramin is reported to be located in the nucleus of cells exposed to suramin (Bojanowski *et al*, 1992). In the present study, we demonstrated that suramin suppressed DNA-PK activity and DSBs repair *in vivo*. Our present observations suggest that suramin may possibly result in sensitisation of cells to ionising radiation by inactivation of DNA-PK and the impairment of DSBs repair.
